# Compact Four-Port Circularly Polarized MIMO X-Band DRA

**DOI:** 10.3390/s22124461

**Published:** 2022-06-13

**Authors:** Ahmed A. Ibrahim, Hijab Zahra, Syed Muzahir Abbas, Mohamed I. Ahmed, Gaurav Varshney, Subhas Mukhopadhyay, Abdelhady Mahmoud

**Affiliations:** 1 Electronics and Communications Engineering Department, Minia University, El-Minia 61519, Egypt; ahmedabdel_monem@mu.edu.eg; 2 School of Engineering, Faculty of Science and Engineering, Macquarie University, Sydney, NSW 2109, Australia; hijab.zahra@students.mq.edu.au (H.Z.); subhas.mukhopadhyay@mq.edu.au (S.M.); 3 Microstrip Department, Electronics Research Institute, El Nozha, Cairo 11843, Egypt; miahmed@eri.sci.eg; 4 Department of Electronics and Communication Engineering, National Institute of Technology Patna, Patna 800005, India; gauravnitd@outlook.com; 5 Faculty of Engineering, Benha University, Benha 13511, Egypt

**Keywords:** antenna, circular polarization, dielectric resonator, MIMO, X-band

## Abstract

A circularly polarized (CP) multi-input multioutput (MIMO) dielectric resonator (DR) antenna (DRA) with compact size and four ports is implemented. CP radiation was achieved using the deformed DR geometry excited with aperture coupled feeding. A CPDRA with a single and two ports is investigated. The defected ground structure (DGS) was incorporated into the antenna for improving the isolation between the ports. The DGS was incorporated in such a way that the required phase difference between the generated orthogonal degenerate modes is preserved. This concept could be utilized in implementing a compact four-port CP antenna. The MIMO antenna provides a 10 dB impedance bandwidth of 38% (8.5–12.5 GHz) and a 3 dB AR bandwidth of 9.32% (9.2–10.1 GHz). The gain of the implemented antenna was around 6 dBi in the band where CP radiation was achieved. The MIMO performance parameters were calculated, and their values remained within the acceptable limits. The implemented antenna could suitably be used in X-band applications.

## 1. Introduction

The dielectric resonator (DR) antenna (DRA) is becoming the best choice over other antennas in high-frequency wireless systems due to its advantages in providing high radiation efficiency and gain with low losses [1,2]. In recent days, techniques have been implemented for developing multiport antennas and DRAs [3]. The usage of multiport antennas enables wireless systems with a higher data transfer rate [4]. Several multiport DRAs offering different characteristics have recently been implemented [5,6,7,8,9]. The literature shows that the latest issues in multiport DRAs are (i) implementing the technique of the generation of circularly polarized (CP) fields [5], (ii) improving the isolation between the ports [10], and (iii) increasing the number of ports by maintaining the antenna size compact [11]. Researchers are working towards finding the best solutions for all these research issues. In the current scenario, implementing the CP multiport antenna is a challenging task because (i) the generation of CP fields needs the excitation of orthogonal degenerate modes with quarter-phase difference, which becomes difficult to maintain in the case of multiport antenna systems [10,12,13]; and (ii) increasing the number of ports becomes difficult because the isolation needs to be maintained within the acceptable limits for an antenna system along with the CP fields [7,10,12,14]. Moreover, the advancement of small-scale systems needs the compact size of a multiport antenna [11,15]. Very few research works have reported on these research issues [7,8,10,12,14,16]. For implementing a CP MIMO antenna, researchers are following the procedure (i) utilizing any CP generation technique in a single-port antenna, and (ii) implementing the compact multiport antenna structure with high isolation along with the desired phase difference between the generated orthogonal modes, so that CP field can be obtained. A traditional cross-slot is investigated for fulfilling the aim of achieving the CP response in MIMO DRA with two ports [7]. A multiport DRA with two ports used metal strips to achieve a CP field [10]. A CP multiport DRA was reported with the deformed geometry of the DR [16]. Moreover, a research work was reported with the usage of a quarter modified feedline is a two-port CP MIMO DRA [12]. Another research work utilized a traditional stair-shaped slot for excitation in implementing a four-port MIMO antenna [13]. These research works utilized the techniques of obtaining the CP response in DRA, which had earlier been reported in the case of a single-port antenna; here, the MIMO antenna was implemented with the said objective. Still, most of all these recently reported antenna structures can provide the facility up to only two ports.

To find a solution to the stated research issues, a compact four-port CP MIMO DRA was designed and implemented in this research work. Utilizing a deformed geometry of the DR in implementing the CP antenna or antenna array is always reliable over deforming the feeding line from the point of structural stability [17,18,19]. So, a deformed geometry of the DR is used for obtaining the CP field. TAantenna analysis with a single radiating element was conducted to obtain the radiation behavior of the antenna.

This study was utilized in the implementation of a two-port MIMO antenna with CP response by the appropriate incorporation of DGS. The concept of a two-port MIMO antenna is utilized in implanting a four-port CP MIMO DRA. The four radiating elements are arranged with the simple aperture coupled feeding into a compact space. The defected ground structure was incorporated into the antenna structure to enhance the isolation between the radiators, so that the phase differences between the orthogonal modes required to achieve the CP response are preserved. The suggested antenna could suitably be used in X-band applications with 38% (8.5–12.5 GHz) and 9.32% (9.2–10.1 GHz) impedance bandwidth and AR bandwidth, respectively. The deformed geometries of the DR with a single port were previously reported [20,21,22,23]. However, implementing the four-port MIMO antenna with CP response has not been investigated till now, and this is the novelty of this research work. Providing the CP response in a compact antenna structure with an increased number of ports is the advantage of the implemented antenna. The novelty and advantages of the reported research work can be summarized in the following points.

- The compact structure of a four-port CP MIMO DRA with the utilization of the deformed shape of the DR and DGS together. Implementing a four-port CP MIMO DRA with high isolation in a compact geometry with the utilization of DGS is a novel technique proposed in the reported research work. Obtaining the compact geometry of the four-port CP MIMO DRA is not available in the literature.
- A technique of obtaining the high isolation between the ports along with the CP response and maintaining the four ports in a compact geometry of antenna.
- High gain is another advantage of the proposed antenna structure that is generally difficult to obtain in the case of C.
- PDRA.

## 2. Single-Element Analysis

**Figure 1:**
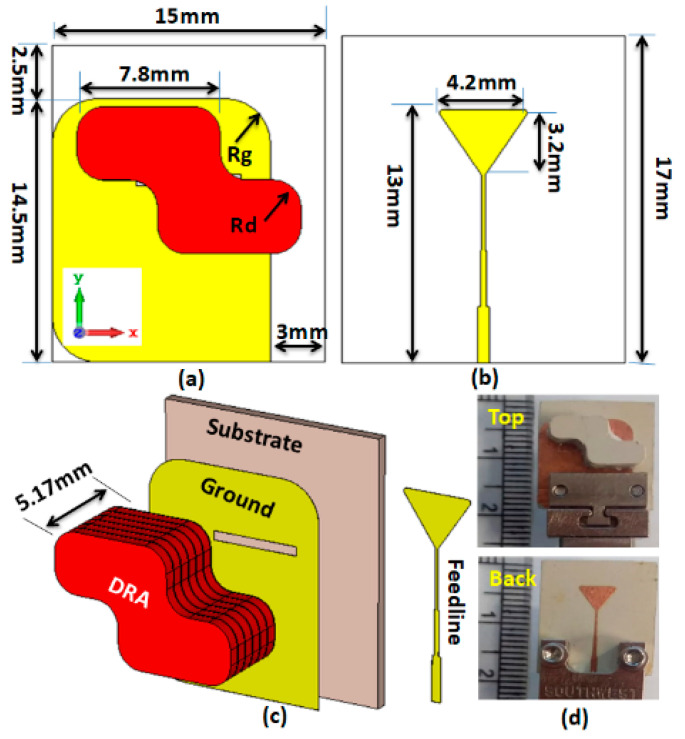
Single-element DRA layout: (**a**) 2D front view; (**b**) 2D back view; (**c**) 3D view; (**d**) fabricated prototype with single DR element.

Figure 1 illustrates the 2D and 3D layout of the CPDRA. The antenna contained a DR of deformed shape, so that the CP field could be obtained as shown in Figure 1. The simplest deformed shape of the DR that is generally used in obtaining the CP response is the stair-shaped geometry [20,22,24]; so, this was also utilized here to fulfil the aim of this research work, i.e., implementing a compact CP MIMO antenna. The DR was composed of material with a permittivity of 10.2, and added above the substrate of the thickness of 0.762 mm. The dimensions of the DR could be selected using the dielectric waveguide model (DWM) by considering the rectangular geometry, and can then be deformed for obtaining the desired polarization and response [23,25]. The simulation was performed by considering the fabrication tolerances, as the prototype was fabricated with multiple layers of dielectric material. The power was coupled to the DR using a rectangular slot with dimensions of 5.8 × 0.6 mm in aperture. A 50 Ω microstrip line of 0.6 mm width was used as the transmission line. The microstrip line was terminated with a λ/4 triangular transformer stub for obtaining an appropriate impedance matching. The proposed DR and the ground had a curvature with a radius $R_{d}=1.5\;\mathrm{mm}$ and $R_{g}=2.5\;\mathrm{mm}$. The reason for chamfering the ground plane and reducing its dimensions was for preserving the isolation between ports, and keeping the desired phase differences between the orthogonal modes to generate the CP field. These values were optimized to achieve the desired frequency response.

**Figure 2:**
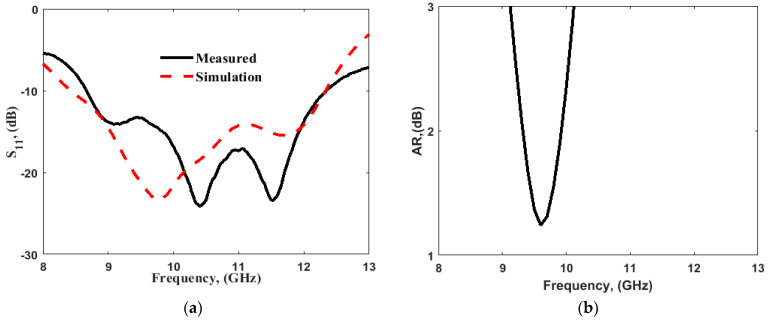
(**a**) Simulated and measured S-parameter response; (**b**) AR for single-element DRA.

**Figure 3:**
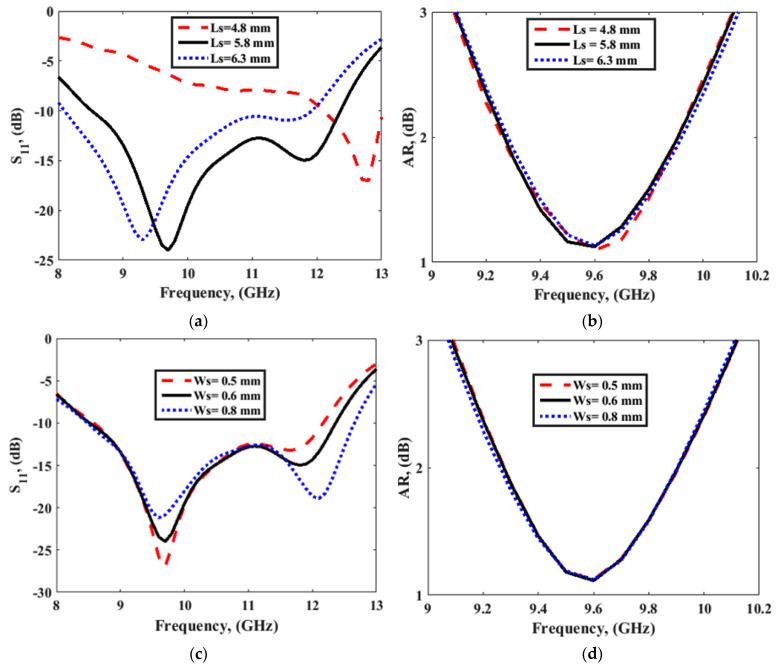
Effect of parameters $L_{s}$ on (**a**) S_11_ and (**b**) AR and the effect of $w_{s}$ on (**c**) S_11_ and (**d**) AR, for single-element DRA.

**Figure 4:**
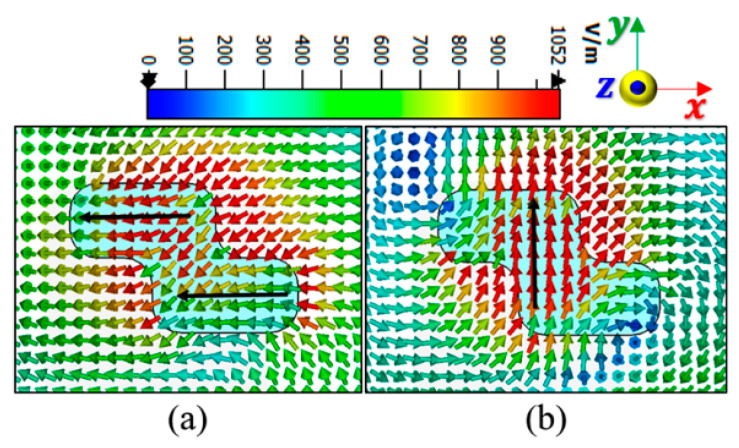
E-field distribution in z = 5.17 mm, z=5.17 mm plane: (**a**) 9.64 and (**b**) 11.88 GHz.

**Figure 5:**
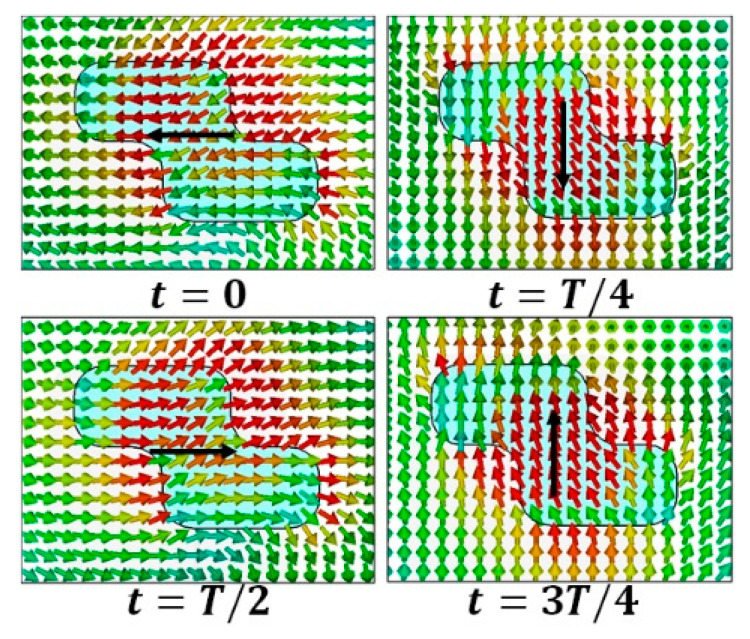
E-field distribution in z = 5.17 mm, z=5.17 mm plane at frequency of 9.77 GHz at the different time instants.

This antenna was fabricated, and the results obtained from the simulation using CST microwave studio were validated by the measurement as illustrated in Figure 2. The antenna displayed an impedance bandwidth of 38% (8.5–12.5 GHz), along with the 3 dB AR passband extended from 9.1 to 10.1 GHz offering 10.4% AR bandwidth. The axial ratio bandwidth was not dependent upon impedance matching and was related to the orthogonal modes [10,12,13]. The DWM model does not consider the effect of the type of feed; hence, the effect of variation in the dimensions of the coupling slot is reported in Figure 3, so that one can infer the conclusions for obtaining the desired response by maintaining the phase difference between the generated orthogonal modes. Figure 4a,b show the E-field distribution at the top of the DR in a z=h plane at frequencies of 9.64 and 11.88 GHz, respectively. The field distribution in the antenna corresponded to modes ${\mathrm{TE}}_{11\delta }^{y}$ and ${\mathrm{TE}}_{11\delta }^{x}$ at frequencies of 9.64 and 11.88 GHz, respectively. This confirmed the generation of two orthogonal degenerate modes, which is required for obtaining the CP field. These modes provide circular polarization at a frequency of 9.77 GHz. The field was drawn at a frequency of 9.77 GHz at different time instants, as shown in Figure 5. The field vectors rotated in the counterclockwise direction, confirming the generation of the dominant right-hand CP field, which was later confirmed during the study of the radiation pattern.

## 3. Two-Port MIMO Antenna

**Figure 6:**
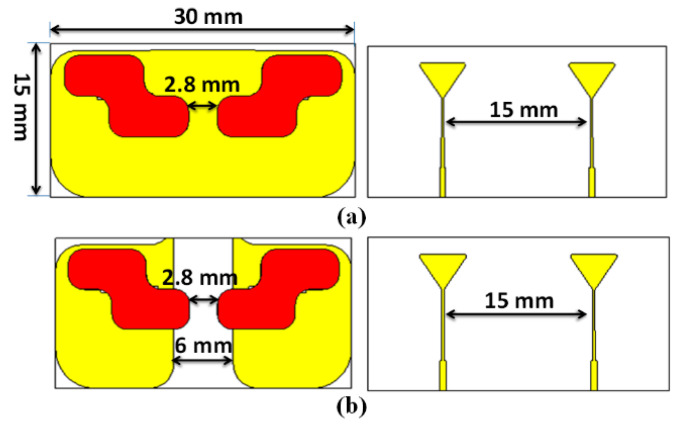
Two-element CP MIMO DRA (**a**) without and (**b**) with DGS.

**Figure 7:**
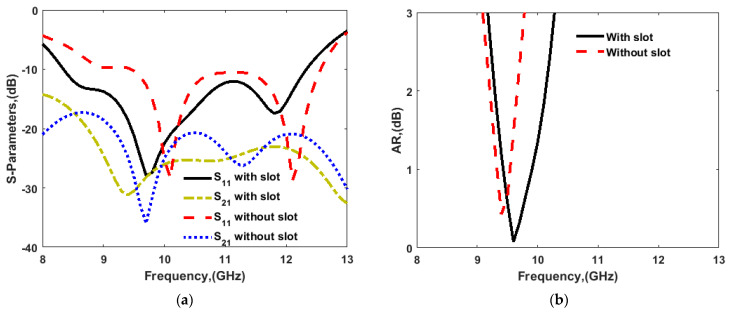
(**a**) S-parameters and (**b**) AR response of two-port antenna with and without slots in the ground plane.

A two-port CP MIMO DRA was designed as shown in Figure 6. Two structures were investigated: one with the full ground, and another with defected ground structure (DGS), as shown in Figure 6a,b, respectively. Figure 7 shows the frequency response of the S11 parameter and AR in both cases. Table 1 shows the performance of both antennas. As usual, the DGS structure enhanced the impedance bandwidth and improved the isolation between ports. Furthermore, the appropriate incorporation of slots in the ground plane improved the AR bandwidth. The phase differences between orthogonal modes could be preserved at the required limit with their desired magnitude; hence, the AR bandwidth was improved after the incorporation of the DGS. Here, in two-port antennas, the edge-to-edge separation between the DR elements was kept as 2.8 mm (0.08$\lambda_{o}$ at 8.5 GHz) with a center to the center separation of 15 mm (0.4$\lambda_{o}$ at 8.5 GHz).

**Figure 8:**
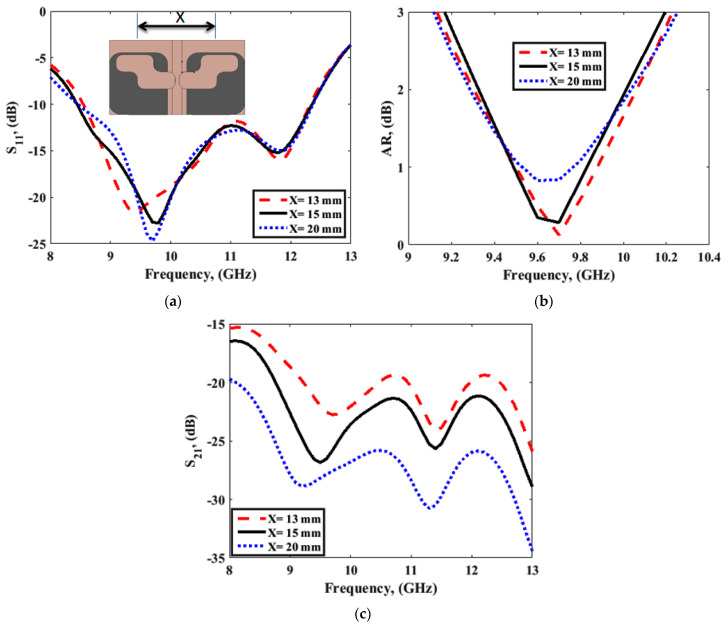
(**a**) S_11_, (**b**) AR, and (**c**) S_21_ parameter with separation between the DR antennas with a slot in the ground.

Increasing the distance between antenna elements being represented by X, the mutual coupling decreased, but the size of the antenna increased. The circular polarization at the proposed band of the antenna was affected by keeping the radiators close to each other. So, the distance between antenna elements was optimized to achieve low mutual coupling with compact size, and maintain the circular polarization. Figure 8a–c present the S11, AR, and S21, respectively, corresponding to various separation distances among the two-element DRAs. The optimized distances among DRAs were chosen on the basis of a parametric study; Figure 8 shows that the optimized separation distance as X = 15 mm provided a better 3 dB.

## 4. Four-Port CP MIMO DRA

**Figure 9:**
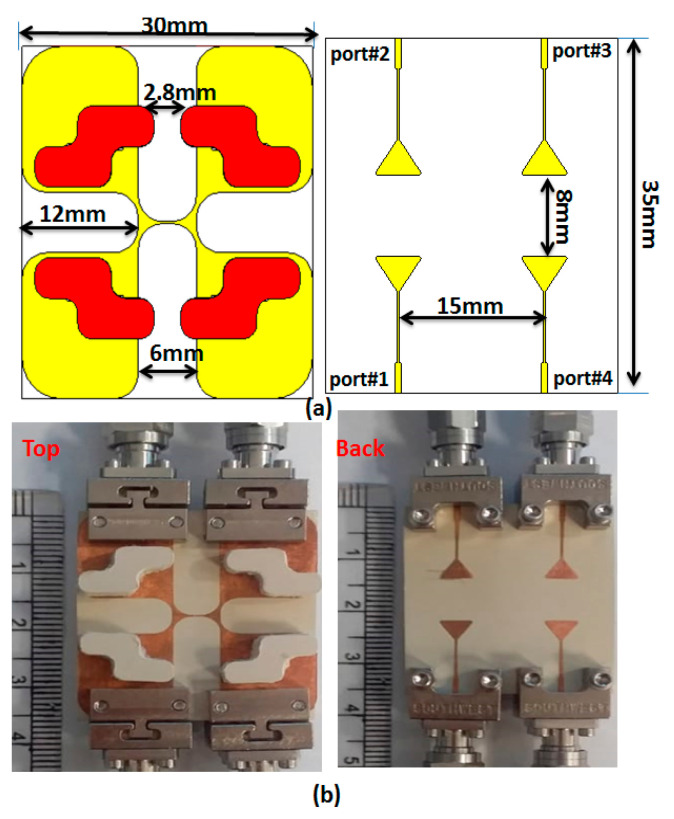
Proposed four-port DRA layout: (**a**) 2D front/back view; (**b**) photo of the fabricated model.

Analysis of a two-port CP MIMO antenna revealed that a four-port CP MIMO DRA could also be implemented as shown in Figure 9. As the use of DGS improves antenna performance, it was applied to the final antenna structure. The four antennas shared the same ground to reduce the antenna size into a compact 30 × 30 mm^2^. The symmetric antenna structure provided similar results at Ports 1 and 3, and Ports 2 and 4. The location of the DR was selected so that the proposed antenna could maintain the high isolation between the ports, and the phase difference between the generated orthogonal modes remained intact. The structure of a two-port antenna was repeated by maintaining the separation of around a quarter wavelength, so that the desired results could be obtained without changing the basic nature of radiation of the single-port antenna.

**Figure 10:**
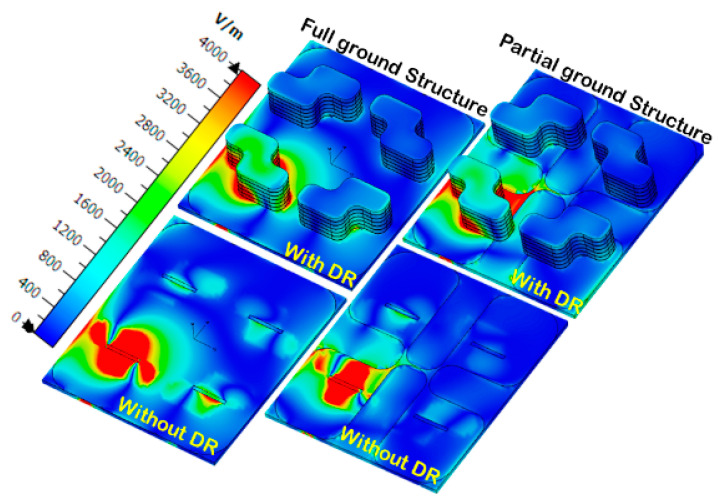
E-field distribution in four-port antenna with full and partial ground structure at 9.53 GHz.

**Figure 11:**
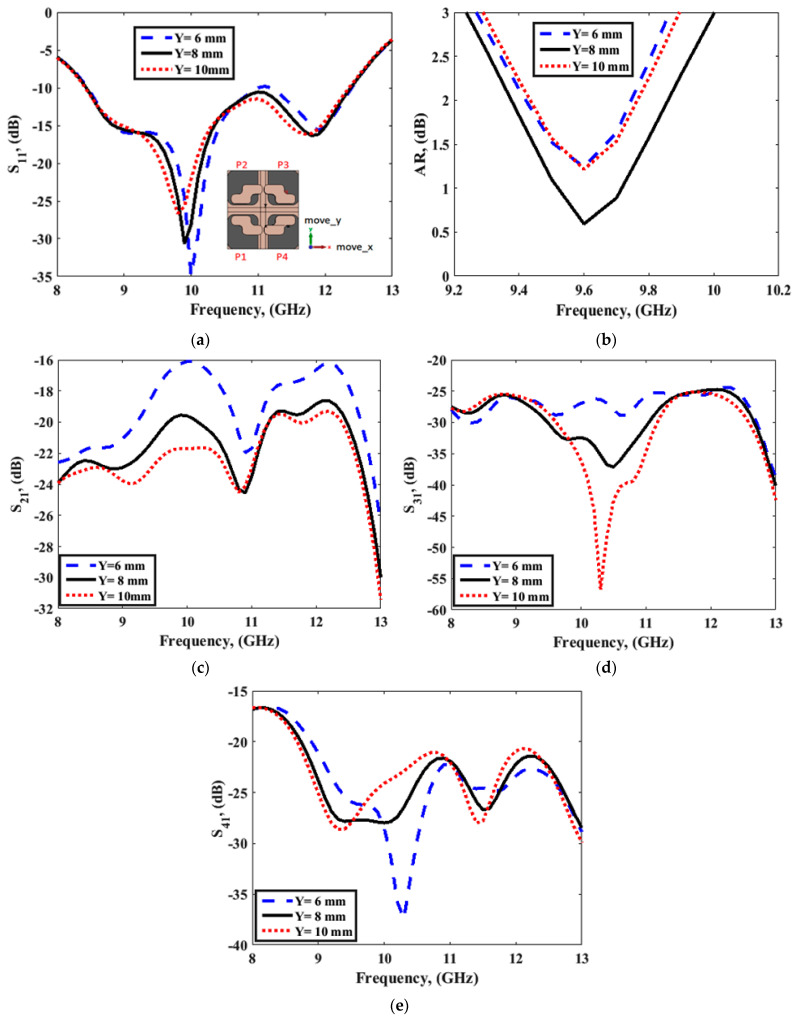
(**a**) S_11_ parameter, (**b**) AR response, and (**c**–**e**) isolation between ports for 4-port MIMO configuration.

**Figure 12:**
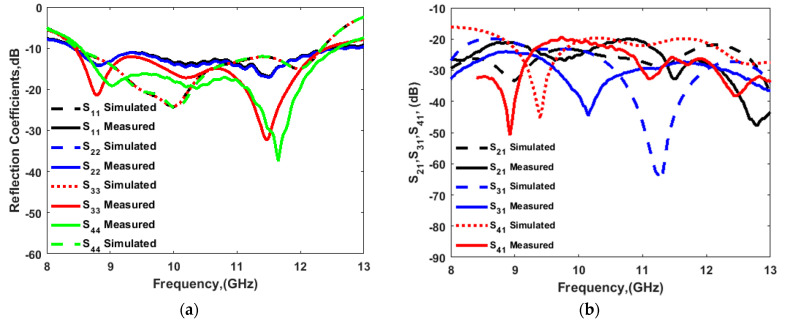
Measured and simulated (**a**) reflection and (**b**) isolation response for 4-port MIMO configuration.

**Figure 13:**
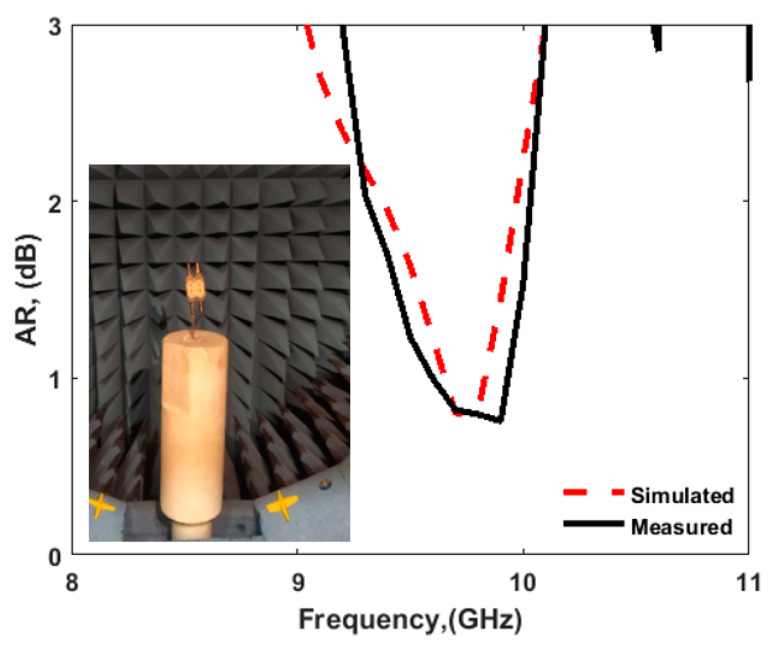
AR response of four-port CP MIMO DRA (simulated and measured).

Figure 9b shows the fabricated prototype of the CP MIMO DRA. Figure 10 shows the electric field distribution in the antenna structure without and with DGS. The field distribution shows the coupling between the adjacent radiating DR elements in a case when four ports were applied. Figure 10 also shows that there was high coupling in the case of usage of the full ground structure. The usage of DGS improved the isolation between ports. The color bar shows that the red field did not appear in the second slot area when DGS was applied. Especially at lower frequencies, this coupling was stronger in the case of a four-port structure in comparison to the two-port structure shown in Figure 6. At higher frequencies, the spacing between radiating elements became comparable to the half-wavelength; hence, the isolation was not much affected. The S-parameter response in these two cases was expected to be as usual, so it is not reported in this manuscript. The field distribution is only reported at lower frequencies and could be assumed at higher frequencies. The four-port antenna contained four radiating elements. The effect of horizontal separation between the radiating elements of the antenna having two ports was observed earlier in this manuscript (Figure 8). The effect of vertical separation being represented by Y is shown in Figure 11a–e. The variation in Y did not significantly affect the reflection coefficient. The variation in Y may affect the phase difference between the generated orthogonal modes desired for generating the CP response; thus, the AR response was varied. The increment in Y enhanced the isolation between radiating elements, which were separated by Y but not by X, as expected. The port parameters were measured using vector network analyzer ZVA 67 to validate the simulated results, as shown in Figure 12. The variation in the S parameter response at each port was due to the fabrication tolerances. A 10 dB bandwidth of 38% (8.5–12.5 GHz) was obtained from the antenna. The isolation between ports is illustrated in Figure 11e. The measured isolation results had a level higher than 20 dB in the operating band. The simulated and measured results of 3 dB AR are displayed in Figure 13. The antenna had a bandwidth of 10.41% (9.1–10.1 GHz) and 9.3% (9.2–10.1 GHz), respectively.

**Figure 14:**
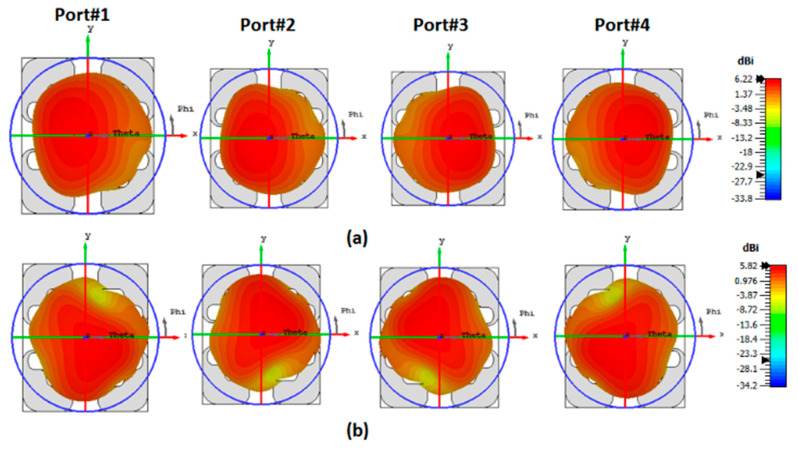
Simulated 3D realized gain of four-port DRA at (**a**) 9 GHz and (**b**) 11 GHz.

**Figure 15:**
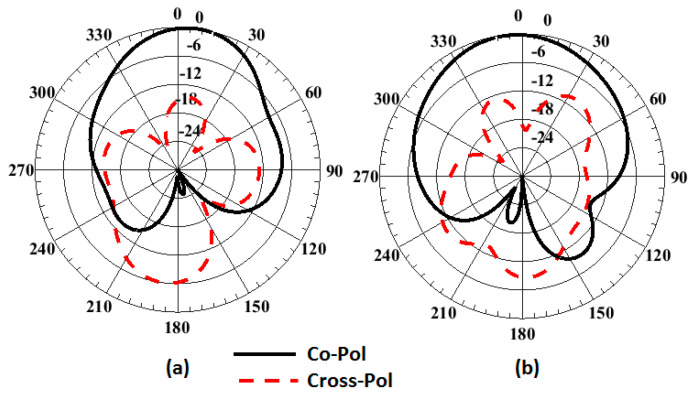
Simulated field pattern of the four ports DRA at 9.77 GH in (**a**) φ = 0° and (**b**) φ = 90° plane (with the applied input at Port 1 and keeping the other terminated with the matched load).

**Figure 16:**
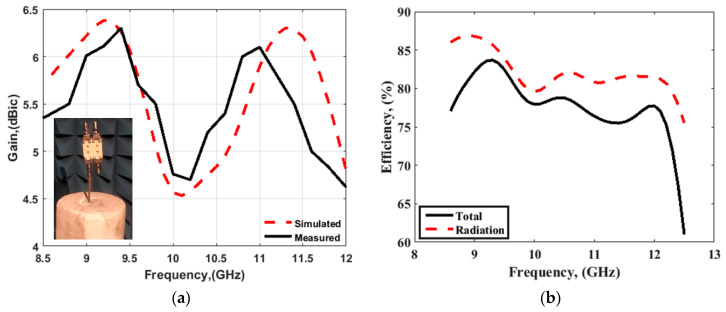
(**a**) Peak gain and (**b**) efficiency of proposed four-port DRA.

**Figure 17:**
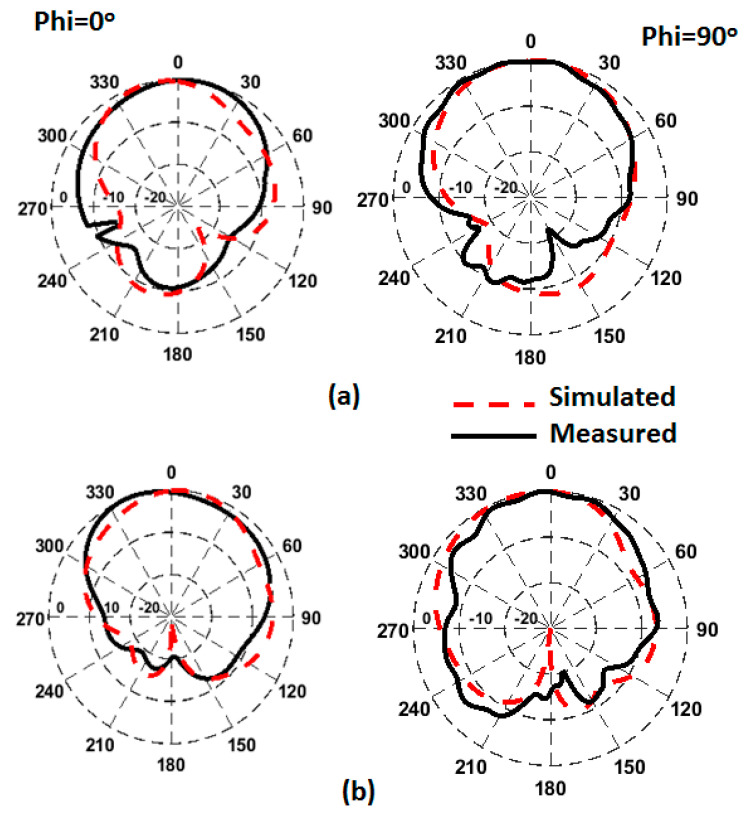
Radiation patterns of four-port DRA (**a**) at 9 (**b**) at 11 GHz.

The mismatching between the two results was due to fabrication tolerances, but it was still at a good level. The advantage of the proposed CP MIMO DRA is that it provides the CP response at all individual ports. Figure 14 illustrates the simulated 3D patterns of the MIMO DRA at each port at 9 and 11 GHz. As the antenna operated with the fundamental mode, the broadside radiation was obtained. Figure 15 shows the simulated RHCP and left-hand (LH) CP field pattern at the frequency where the dip of AR was obtained. The antenna provided the dominant LHCP by around 18 dB over the cross-polarized component in the direction of radiation, which remained broadside. Gain (simulated and measured) and efficiency (simulated) results are shown in Figure 16. The antenna provided a gain of more than 4.5 dBic over the passband with each port. The radiation efficiency of the antenna also remained at more than 80% in the operating passband. The simulated and measured radiation pattern results of the suggested antenna are illustrated in Figure 17. Far-field measurement was conducted using the Starlab system. The envelope of the simulated far-field response matches well with the measured one.

## 5. MIMO Performance Analysis

**Figure 18:**
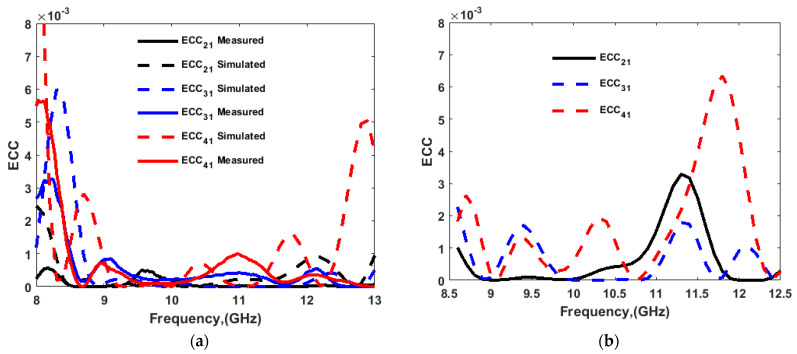
ECC (**a**) measured and simulated from S-parameters; (**b**) from far-field.

**Figure 19:**
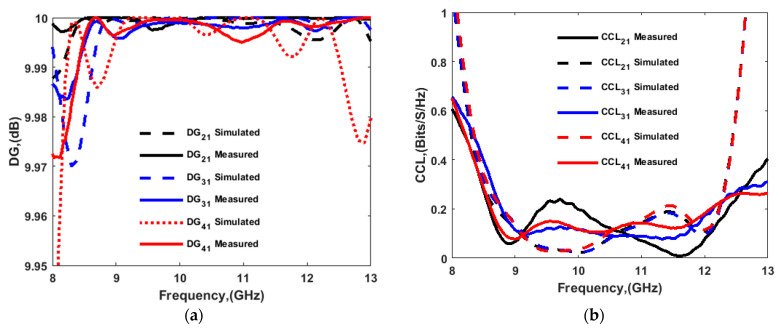
(**a**) Diversity gain (DG) and (**b**) channel capacity loss (CCL).

**Figure 20:**
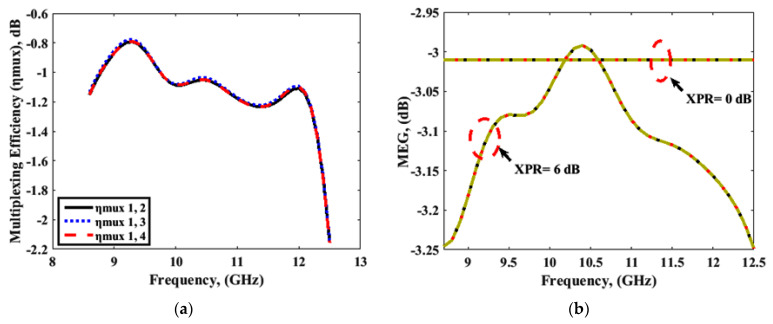
(**a**) Multiplexing efficiency ($\eta_{mux}$), and (**b**) mean effective gain (MEG).

**Figure 21:**
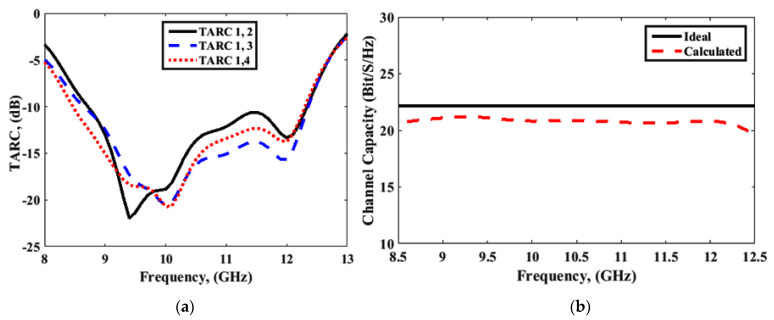
(**a**) Total active reflection coefficient (TARC); (**b**) channel capacity.

The diversity characteristics and performance of the MIMO system could be examined by using the envelop correlation coefficient (ECC), diversity gain (DG), channel capacity loss (CCL), multiplexing efficiency ($\eta_{mux}$), mean effective gain (MEG), total active reflection coefficient (TARC), and channel capacity. ECC, DG, and CCL were calculated using the simulated and measured port parameters, and the simulated far-field, and the results are shown in Figure 18 and Figure 19, respectively. Results for $\eta_{mux}$, MEG, TARC, and channel capacity were calculated using simulated parameters only, and are presented in Figure 20 and Figure 21. The MIMO performance parameters remained within the acceptable limits, proving that the proposed antenna is a good candidate for CP MIMO operation [16]. The calculation method of the parameters is already available in the literature, and a similar method was followed [15,16,26,27]. Table 2 shows a comparison of the proposed antenna with others available in the literature [10,12,13,14,16,25,28,29,30]. The comparison table shows the following. (i) Many other antennas are available with the two-port geometry, and the number of ports of the proposed antenna was doubled. (ii) The size of the antenna was significantly small if DGS was applied in the structure by maintaining high isolation. (iii) The gain of the proposed antenna was higher or comparable with the other four-port antennas. The CP bandwidth was also comparatively wider in the case of a four-port DRA.

## 6. Conclusions

A four-port CPDRA with compact size was designed and implemented. CP radiation was achieved using the deformed shape of the DR excited with aperture coupled feeding. The study of the single-port CPDRA revealed that a two-port CP antenna could be implemented if a DGS is properly incorporated into the antenna structure. This concept was also applied to implementing a four-port CP antenna. A 10 dB impedance bandwidth of 38% (8.5–12.5 GHz) with a 3 dB AR bandwidth of 9.32% (9.2–10.1 GHz) was achieved. In addition, a gain of 6 dBi was obtained in the band where CP radiation was achieved. The MIMO performance parameters were calculated, and their values remained within the acceptable limits. The suggested antenna could suitably be used in X-band applications.

## Figures and Tables

**Table 1:** Performance of a two-port antenna.

Antenna	BWIM (%)/(GHz) (S11 < −10 dB)	Minimal Isolation (dB)	BWAR/(GHz) (AR < 3 dB)
Without DGS	31.96 (9.2–12.7)	20–35	7.40 (9.1–9.8)
With DGS	38.09 (8.5–12.5)	20–35	11.28 (9.2–10.03)

**Table 2:** Comparison with other CP MIMO DRAs.

Ref	No. of Ports	$f_{r}(\mathrm{GHz})/\mathrm{BW}\;(\%)$	AR BW (GHz)/(%)	Gain (dBi)	Isolation (dB)	ECC	Antenna Size (mm × mm)/λ_0_^2^
[14]	2	3.45/23.1	3.34–4.02/18.5	4.83	>26	˂0.02	95 × 49.7/1.09 × 0.56
[16]	2	6.44/36.7	7.72–8.08/4.55	3.8	15	˂0.05	80 × 80/1.7 × 1.7
[13]	4	3.58/13.1	3.54–3.74/4.9	5.2	>18	0.03	80 × 80/0.95 × 0.95
		5.25/6.09	5.02–5.18/2.3	5.5	20		
[10]	2	4/38.5	3.58–4.40/20.8	6.5	>28	˂0.04	350 × 350/4.3 × 4.3
[25]	2	5.62/21.4	5.15–5.88/13.2	4.7	>18	˂0.05	50 × 50/0.92 × 0.92
[12]	2	3.42/19	3.425–3.6/5	6.8	16.5	˂0.05	110 × 50/1.25 × 0.56
		5.45/9.4	5.45–5.55/2	4.6	16.2		
[28]	4	3.5/14.4	3.34–3.54/5.8	4.2	>15	˂0.5	66 × 60/0.76 × 0.70
[29]	4	4.9/5.1	-	6.2	25	0.002	140 × 45/2.28 × 0.73
[30]	4	5.7/10.5	-	5	18	0.25	30 × 30/0.57 × 0.57
This work	4	10.5/38	9.2–10.1/9.32	6	>22	˂0.005	35 × 30/1.05 × 1.22
